# P-422. Endotracheal Aspirate Gram Stain: Correlation with Semi-quantitative Culture Results

**DOI:** 10.1093/ofid/ofaf695.638

**Published:** 2026-01-11

**Authors:** Talla Bitar, Kevin Lloyd, Aimee Dassner, Monika Geslak, Benjamin M Liu, Joseph M Campos, Meghan Delaney, Esther Esadah, Craig A Shapiro, Michael Evangelista, Joyce Granados, Rachel Strength, Rana F Hamdy

**Affiliations:** Childrens National Hospital, Arlington, TX; Childrens National Hospital, Arlington, TX; Children's National Health System, Washington, District of Columbia; Children's National Hospital, Washington, District of Columbia; Children's National Hospital /George Washington University, Washington, District of Columbia; Children's National Hospital, Washington, District of Columbia; Children's National Hospital, Washington, District of Columbia; Children's National Hospital, Washington, District of Columbia; Children's National Hospital, Washington, District of Columbia; Children's National Hospital, Washington, District of Columbia; Children's National Hospital, Washington, District of Columbia; NIH/NIAID/Children's National Hospital, SIlver Spring, MD; Childrens National Hospital, Arlington, TX

## Abstract

**Background:**

Endotracheal aspirate (ETA) cultures are commonly used in tracheostomy-dependent and mechanically-ventilated children with suspected lower respiratory tract infections. Gram stains of the ETA are performed before culture incubation to assess for white blood cells and microorganisms. Samples without microorganisms on Gram stain typically have low culture yield and rarely grow clinically relevant organisms. Despite this, samples with negative Gram stains are often still processed for culture.

The objective is to assess the correlation between ETA Gram stain and semi-quantitative culture results at a free-standing children’s hospital.

**Figure:**
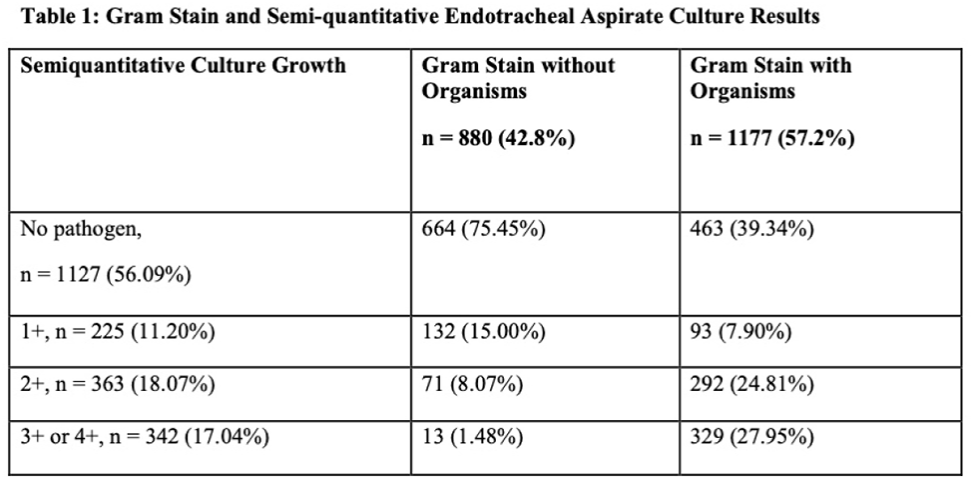
Gram Stain and Semi-quantitative Endotracheal Aspirate Culture Results

**Methods:**

This is a retrospective cohort study of ETA cultures processed at the Children’s National Hospital (CNH) Microbiology Laboratory from 01/2023 to 12/2024. ETA Gram stains and semi-quantitative culture growth (1+, 2+, 3+, or 4+) were reviewed. For cultures that grew more than one organism, the organism with the highest semi-quantitative growth category was used for the sample. Culture growth of 3+ or 4+ was considered substantial.

**Results:**

A total of 2057 samples were included. 1177 (57.2%) had organisms identified on Gram stain, which corresponded with 3+ or 4+ pathogenic growth in 329 (27.9%) samples (Table 1). Of the 880 (42.8%) samples without microorganisms detected on Gram stain, 867 (98.5%) resulted in no substantial bacterial growth; 13 (1.5%) had substantial growth. Using chi-square, we found Gram stain results to be significantly associated with culture results (p< 0.0001). The sensitivity of a Gram stain with microorganisms for positive culture was 96.2% with an associated negative predictive value of 98.5%. Specificity of Gram stain with microorganism was 50.6% with an associated positive predictive value of 28%.

**Conclusion:**

ETA Gram stain with microorganisms is strongly correlated with semi-quantitative respiratory culture results. Despite low specificity and positive predictive value, the utility of Gram stains for ETA samples lies in the sensitivity and negative predictive value of this test for determining the need for subsequent ETA culture.

**Disclosures:**

Rachel Strength, MD, Dexcom: Stocks/Bonds (Public Company)|Johnson and Johnson: Stocks/Bonds (Public Company)|Merck: Stocks/Bonds (Public Company)|Thermo Fisher: Stocks/Bonds (Public Company)|Vertex Pharmaceuticals: Stocks/Bonds (Public Company) Rana F. Hamdy, MD, MPH, MSCE, FPIDS, No company, but co-inventor on a pending patent for StrepApp: co-inventor on a pending patent for StrepApp

